# Sociodemographic characteristics, riding behavior and motorcycle crash involvement: a structural equation modeling approach

**DOI:** 10.5249/jivr.v15i1.1784

**Published:** 2023-01

**Authors:** Sara Naderpour, Seyed Taghi Heydari, Kamran Bagheri Lankarani, Seyed Abbas Motevalian

**Affiliations:** ^*a*^ Department of Epidemiology, School of Public Health, Iran University of Medical Sciences, Tehran, Iran.; ^*b*^ Health Policy Research Center, Institute of Health, Shiraz University of Medical Sciences, Shiraz, Iran.; ^*c*^ Research Center for Addiction and Risky Behaviors (ReCARB), Psychosocial Health Research Institute (PHRI), Iran University of Medical Sciences, Tehran, Iran.

**Keywords:** Structural Equation Modeling, Traffic Safety, Motorcycle Rider, Traffic Errors, Traffic Violations

## Abstract

**Background:**

The increasing rate of traffic crashes involving motorcyclists have turned into a public health and road safety concern. Furthermore, riding behaviors and their precedent factors have been identified as potential determinants for assessing, intervening, and prevent-ing traffic injuries of motorists. This study aimed to identify the effects of a set of demographic and motorcycle-related variables as potential predictors on collision through riding behavior components.

**Methods:**

The study sample was 1,611 motorcyclists who were selected through time-location sampling method from three cities in Iran. They responded a Motorcycle Rider Behavior Questionnaire (MRBQ) and a general questionnaire including sociodemographic and riding-related items. The chosen method to analyze the data was Structural Equation Modeling (SEM) through Lavaan package version 0.6-8 of R software version 4.1.0.

**Results:**

All participants were male (100%) with a mean age of 28.1(SD=8.5) years. About 24.4% of riders experienced at least one crash during the last year and the majority of riders did not hold a motorcycle license (80.1%). The SEM model showed that riding license (0.06) and fre-quency of riding (0.09) had a direct effect on crash involvement. Some latent variables including speed violation (0.13), stunts (0.11) and traffic violation (0.07) had positive effects and safety violation (-0.07) had a negative effect on crash history. There were indirect effects between age and history of crash mediated by speed violation (-0.04), stunts (-0.04), traffic violation (-0.02) and safety violation (0.01). Also, the indirect effects of riding frequency on crash involvement were mediated by speed violation (0.01), traffic violation (0.006) and safe-ty violation (-0.01).

**Conclusions:**

This study’s main finding is that age and riding frequency are the main variables indirectly af-fecting crash involvement. Therefore, periodic training courses for younger riders is essential in order to decreasing crash involvements.

## Introduction

Road traffic crashes are meaningful economic and public health issue worldwide. Mortality due to road traffic injuries has an upward trend accounts for more than 1.35 million deaths per year globally. An alarming issue is that road traffic crashes will be the fifth leading cause of death by 2030. Statistics approve that pedestrians, cyclists, and motorcyclists are classified as more vulnerable to being involved in traffic collisions compared to other road users because motorcycle riders' safety equipment are weaker than car drivers. According to the World Health Organization (WHO) figures, motorcyclists account for 28% of all deaths worldwide.^1^ As a result, enhancing motorcycle safety is a main concern. Human factor is deemed to be the major contributing factor to collisions. Human element plays a substantial role in road injuries influencing up to 95% of collisions. In contrast, road environment factors and vehicle factors affect 28% and 8%, respectively. Individual and behavioral characteristics of riders have significant role in this regard.^2^ An examination of 220 articles identifying risk factors of casualty following motorcycle crashes by Lin and Kraus revealed that inexperience, risk-taking behavior, speed violation, and deliberate disobedience of traffic law increased the risk of a collision and its potential severity.^3^

Furthermore, traffic errors, control errors, speed violations and performing stunts are some of factors affecting motorcyclists' crash.^4-6^

On the other hand, empirical evidence indicates that some individual variables including male gender, young age, more hours riding, driving following alcohol consumption and low socioeconomic level have an enormous impact on the likelihood of engaging in risky riding behavior and collisions.^4-5,7-8^

Elliotte et al.^4^ developed the first Motorcycle Rider Behavior Questionnaire (MRBQ) using an extensive sample (N=8666) according to a study by Reason et al.^8^ on taxonomy of aberrant driver behavior. According to Reason et al.,^8^ drivers' behavior related to road injuries has been dichotomized broadly into "Errors" as "the failure of planned actions to achieve their intended consequences" and "Violations" as "deliberate deviations from those practices believed necessary to maintain the safe operation of a potentially hazardous system". Further studies have also used the MRBQ^3,5-18^ e.g. Motevalian et al.^10^ validated the Persian version of the MRBQ in Iran considering traffic errors, safety violations, traffic violations, stunts, control errors and speed violation. 

According to the massive burden of road traffic injuries and increasing the number of deaths due to motorcyclists, this research tries to identify the effects of demographic traits on road traffic injuries.^19^ The findings of this study can be applied by authorities to develop policies, programs, and relevant strategies to reduce the frequency and magnitude of traffic collisions and increase road safety.

## Methods 

**Participants and procedure**

Data were collected over six months from February 2018 to July 2018. A multi-center cross-sectional research was performed on 1,747 motorcycle riders in Shiraz (n=859), Darab (n=400), and Yazd (n=488) cities in Iran. In this research, a time-location sampling method was used. In the first step, the main streets of cities were identified based on the volume of motorcycle traffic. In the second step, the participants were chosen randomly from motorcyclists who had ridden on these roadways at various times. After obtaining informed consent, trained interviewers completed questionnaires for each rider. Ethics approval for this study was obtained from the Iran University of Medical Sciences.

**Materials**

***Motorcycle Rider Behavior Questionnaire (MRBQ):***

The Motorcycle Rider Behavior Questionnaire (MRBQ) contains 43 items that measure aberrant riding behaviors that could be conducted by a motorcyclist. The MRBQ measure has good reliability with Cronbach's alpha coefficients for the five factors ranging from 0.70 to 0.84.^4^

The Persian version of the original MRBQ validated by Motevalian et al. in 2011 was used in the present study. The Persian version of MRBQ has showed 48 items and for each item, the respondents were asked to report the frequency of engagement in each behavior during last year on 5-point Likert scale (1=never, 2=hardly ever, 3=occasionally, 4=quite often, and 5= nearly all the time). The Principal Components Analysis (PCA) of the revised MRBQ identified six factors consisting of “Speed Violations”, “Traffic Errors”, “Safety Violations”, “Traffic Violations”, “Stunts” and “Control Errors”. Cronbach’s alpha was between 0.79 to 0.91 for each of these factors.^10^

In the present study, we assessed reliability and validity of the Persian version of MRBQ once again. The overall Cronbach’s alpha coefficient was 0.93 indicating a good reliability of the questionnaire. Also Confirmatory Factor Analysis (CFA) was conducted to examine the validity of the questionnaire and it showed a good internal consistency. We dropped six items with lower loading factor including stunts (item 27 and 28), traffic error (item 44), safety violation (item 46), control error (item 47 and 48). Therefore, the final MRBQ consisted of 42 items which was used in the present study. For each of the six factors, the points of loaded items were summed up to calculate the factor score. 

***Demographic, riding and crash history information***

Respondents were also asked to report socio-demographic background e.g. age, education level, marital and employment status, monthly income and their riding information about motorcycle license and riding frequency (days of the week). Participants also provided information on motorcycle crashes (including minor crash) on public roads over the past 12 months. This information was used to calculate the total number of riders' experiences about collision during the last year. 

***Data analysis technique using Structural Equation Model***

We selected the socio-demographic, motorcycling behavior and history of crash involvement to be included in our SEM based on a literature review on road traffic injuries.^3,15,17-18,20^ We assessed 13 variables including age (18-20 years old, 20-29 years old, 30-39, 40-49, and ≤50), marital status (single, married), education level (elementary and junior high school, senior high school and diploma, bachelor degree, and post graduate), occupation (unemployed , employed), income ($140, $140-280, $ 280-560, and $>560), riding frequency (occasionally, 1-3 times a week, 4-6 times a week, and daily), driving license and accident history as observed and independent variables, latent variables (traffic errors, speed violation, safety violation, traffic violation, stunts and control error), and crash experience as observed and dependent variables. 

In order to achieve a model that can measure the direct and indirect effects of demographic variables with the history of crash involvement through the components of motorcycling behavior, the Structural Equation Modeling (SEM) was used. We performed SEM through Lavaan package version 0.6-8 of R software version 4.1.0.

Four goodness-of–fit statistics were used to judge the validity of the estimated model and see how well the data fit with a normal distribution. The Root Mean Square Error of Approximation (RMSEA), were evaluated as absolute fit measures, while the Comparative Fit Index (CFI), TLI and SRMR were taken as incremental fit indexes. As a rule of thumb, a model with CFI and TLI values greater than 0.90 is considered satisfactory, whereas the conventional values of RMSEA and SRMR are below 0.10 for most acceptable model. However, RMSEA below 0.08 would indicate a good fit of the models.^21^

## Results

**Socio-demographic characteristics and riding behavior**

A basic statistical description of the collected sample is presented in Table 1 . A total of 1,747 questionnaires were completed. Following data cleaning, the final sample contained 1,611 motorcycle riders. All participants were male (100%) ranging from 18 to 64 years (Mean±SD: 28.1±8.5) with only a small percentage of riders aged 50 years and older (2.7%). Likewise, most of the riders were single (62.9%), and in respect of education level, senior high school and diploma were frequent (44.8%). The majority of riders did not hold a motorcycle license (80.1%), and on average, the riders who rode daily accounted for large proportions of our sample (57.4%). Furthermore, 24.4% of the sample had been involved in a motorcycle crash when they were riding over the past 12 months. 

**Table 1:** Socio-demographic characteristics and riding behavior.

Variables		N= 1,611	Traffic Errors (11-55)	Speed Violation(11-52)	Stunts(6-30)	Control Errors(5-25)	Traffic Violation(5-25)	Safety Violation(4-20)
			Mean(S.E)	Mean(S.E)	Mean(S.E)	Mean(S.E)	Mean(S.E)	Mean(S.E)
**Age(year)**	18-20	287(17.8)	25.14(0.43)	26.38(0.51)	10.49(0.25)	9.13(0.19)	11.30(0.19)	17.73(0.31)
	20-29	770(47.8)	24.62(0.26)	24.38(0.28)	9.23(0.13)	8.79(0.10)	11.08(0.12)	18.28(0.17)
	30-39	382(23.7)	21.66(0.34)	20.38(0.36)	7.34(0.14)	7.63(0.12)	9.88(0.16)	16.37(0.23)
	40-49	129(8)	20.97(0.60)	18.19(0.55)	6.98(0.21)	7.57(0.23)	8.96(0.26)	14.96(0.45)
	>-50	43(2.7)	19.16(1.08)	16.74(1.02)	5.97(0.31)	6.72(0.44)	7.65(0.45)	15.13(0.62)
**Marital Status**	Single	1013(62.9)	24.48(0.23)	24.55(0.26)	9.34(0.12)	8.67(0.09)	11.10(0.10)	17.93(0.15)
	Married	598(37.1)	22.03(0.29)	20.61(0.30)	7.72(0.13)	7.99(0.11)	9.68(0.13)	16.44(0.20)
**Education Level**	Elementary and Junior high school	187(11.6)	22.17(0.55)	23(0.64)	8.50(0.26)	8.02(0.19)	10.33(0.26)	17.01(0.35)
	Senior high school and Di-ploma	721(44.8)	23.98(0.27)	23.56(0.31)	9.03(0.14)	8.50(0.11)	10.81(0.12)	17.73(0.18)
	Bachelor de-gree	590(36.6)	23.70(0.30)	22.76(0.32)	8.54(0.15)	8.45(0.12)	10.46(0.14)	17.43(0.20)
	Post graduate	113(7)	22.62(0.66)	21.99(0.80)	8.29(0.34)	8.39(0.28)	10.03(0.35)	15.53(0.46)
**Occupa-tion**	Unemployed	62(3.84)	21.59(0.92)	22.75(0.99)	8.50(0.48)	7.77(0.35)	10.11(0.37)	17.37(0.56)
	Employed	1549(96.15)	23.65(0.18)	23.10(0.21)	8.75(0.09)	8.45(0.07)	10.59(0.08)	17.38(0.12)
**Income**	140 $	508(31.5)	24.25(0.33)	24.50(0.35)	9.49(0.17)	8.64(0.13)	10.83(0.14)	17.87(0.22)
	140-280$	321(19.9)	25.42(0.41)	24.90(0.47)	9.30(0.21)	8.90(0.16)	11.05(0.19)	17.81(0.28)
	280-550 $	514(31.9)	22.33(0.30)	21.94(0.35)	8.14(0.15)	8.30(0.12)	10.45(0.15)	16.84(0.22)
	>550 $	268(16.6)	22.45(0.43)	20.45(0.48)	7.79(0.21)	7.66(0.16)	9.76(0.20)	16.96(0.26)
**Riding Frequency**	Occasionally	318(19.7)	21.83(0.42)	20.75(0.43)	8.10(0.20)	8.01(0.17)	9.75(0.20)	15.34(0.27)
	1-3 times a week	150(9.3)	22.05(0.57)	21.88(0.64)	8.75(0.34)	8.17(0.22)	9.96(0.26)	16.16(0.39)
	4-6 times a week	218(13.5)	22.57(0.44)	22.51(0.50)	8.88(0.23)	8.50(0.17)	10.58(0.21)	16.80(0.30)
	Daily	925(57.4)	24.65(0.24)	24.23(0.27)	8.92(0.12)	8.58(0.10)	10.95(0.11)	18.41(0.16)
**Riding License**	No	1291(80.1)	23.65(0.20)	23.27(0.22)	8.76(0.10)	8.34(0.08)	10.56(0.09)	17.70(0.13)
	Yes	320(19.9)	23.25(0.40)	22.35(0.46)	8.63(0.22)	8.73(0.17)	10.63(0.19)	16.10(0.30)
**Crash History**	No	1218(75.6)	22.86(0.20)	21.96(0.22)	8.25(0.10)	8.11(0.08)	10.15(0.09)	17.12(0.14)
	Yes	393(24.4)	25.79(0.36)	26.57(0.41)	10.26(0.20)	9.39(0.15)	11.87(0.17)	18.18(0.25)

Mean and standard error of the six riding behavior factors are calculated for each of the sociodemographic categories. Also the range of factor scores are shown in parenthesis (Table 1 ). 

**Structural Equation Modeling (SEM) analyses by R (Lavaan) program:**

**Figure 1:**
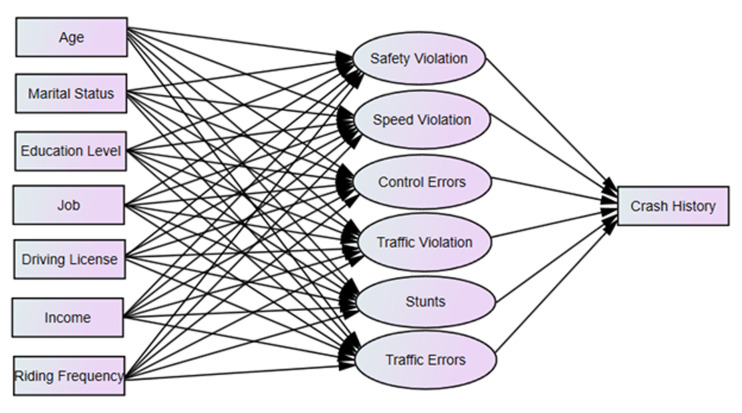
The hypothesized model.

**Figure 2:**
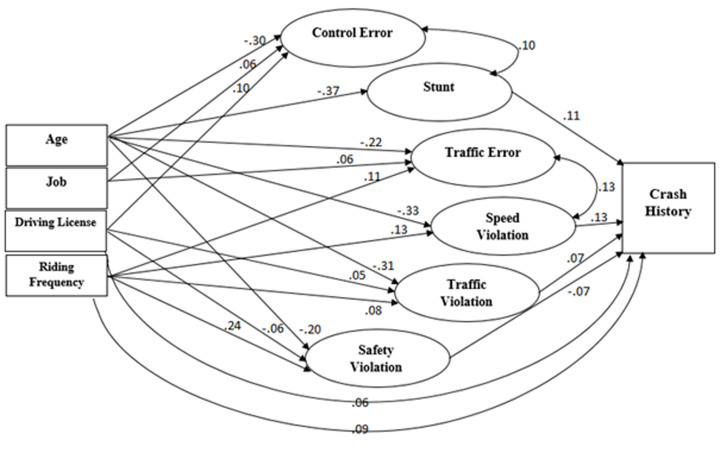
Final model of socio-demographic and riding factors on collisions through motorcycle rider behaviors. Ellipse indicates latent, unobservable constructs; box indicates observed variable; straight line with one arrowhead denotes direct effect; curved line denotes the correlation.

**Figure 3:**
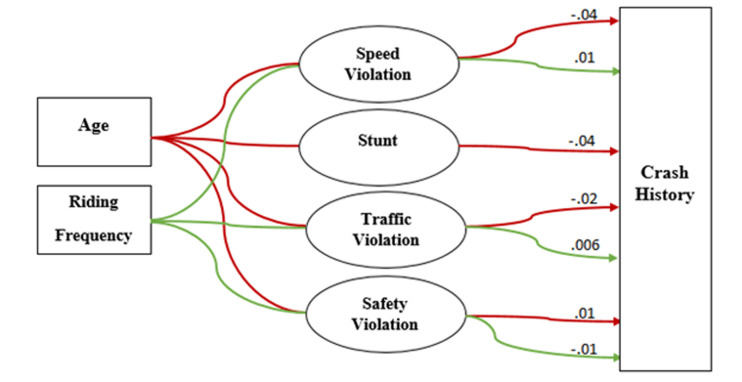
Indirect effects of socio-demographic and riding factors on crash through motorcycle rider behaviors. Ellipse indicates latent, unobservable constructs; box indicates observed variable; curved line with one arrowhead denotes indirect effect.

The hypothesized model didn’t fit the data (RMSEA: 0.20; CFI: 0.10; TLI: 0.05; SRMR: 0.26) (Figure 1). Non-significant paths were removed and a few additional paths were added to improve model fit. We dropped marital status, education level, income as variables and added the following correlations: traffic error and speed violation; stunts and control error; and stunts and traffic violation. All items depicting the latent variables of motorcycle rider behaviors had significant factor loading. The final model had an acceptable fit (RMSEA: 0.07; CFI and TLI ~ 0.65; SRMR: 0.18, Chi-square/degree of freedom= 10.67) and the fully standardized path coefficients are presented in Figures 2 and 3. We reported pathways only for statistically significant standardized path coefficients at the p<0.05 level. 

The two socio-demographic factors; riding license (0.06) and frequency of riding (0.09) deemed to have a direct statistically significant effect on crash involvement. Age had a strong negative effect on all latent variables; traffic error (-0. 22), speed violation (-0.33), stunts (-0.37), control error (-0.30), traffic violation (-0.31) and safety violation (-0.20). Occupation had significant positive effect on both errors; traffic error and control error (0.06). Frequency of riding was found to have positive effect on control error (0.11), speed violation (0.13), traffic violation (0.08), and safety violation (0.24). In addition, driving license had positive effect on control error (0.10), traffic violation (0.05) and negative effect on safety violation (-0.06). 

Regarding the effects of motorcycle rider behavior factors on crashes, latent variables of speed violation (0.13), stunts (0.11) and traffic violation (0.07) had positive effects and safety violation (-0.07) had a negative effect on the history of collisions.

As shown in Figure 3, there are specific indirect effects between age and history of crash mediated by speed violation (-0.04; p≤0.001), stunts (-0.04; p≤0.001), traffic violation (-0.02; p= 0.01) and safety violation (0.01; p= 0.01). We also found indirect effects between riding frequency and crash involvement mediated by speed violation (0.01; p≤0.001), traffic violation (0.006; p= 0.01) and safety violation (-0.01; p= 0.01). 

## Discussion

Based on the response provided by 1,611 motorcyclists taking part in this research, the present study explained the association between self-reported traffic crashes of motorcyclists and demographic characteristics through riding behaviors using the structural equation modeling approach. According to the results, the probability of a crash is influenced indirectly by age and riding frequency. Speed violation, stunt, traffic violation, and safety violation were found to mediate the effects of age on the history of crashes. The age did not have a significant direct effect on the history of crashes, whereas studies^4,22-23^ demonstrated a direct effect of age on crashes. 

Prior research has suggested that lack of proper riding skills or experience combined with a propensity to do risky behaviors may explain the higher risk among young people.^24^ It is appropriate to build more speed bump and tighten control over drunken riders to reduce traffic violation and improve road safety. In respect of the indirect effect of riding frequency on crashes, we found out that speed violation, traffic violation, and safety violation tend to mediate the frequency of motorcycling on the rate of crashes. High frequency of riding per week is associated with a higher rate of crashes through the paths of speed violation and traffic violation; and a lower rate of crashes by the path of safety violation. In addition, the present study showed that the frequency of motorcycling has a direct positive effect on the rate of crashes. These results were consistent with findings from previous research indicating that the number of years of motorcycling is linked to collision involvement.^22,24^

Previous researchers did not examine motorcycling frequency effects on the history of crashes but Fagnant et al discovered that crash rates in the last three years among drivers was positively correlated with working hours per week. This indicates that more hours of driving per week cause fatigue, low attention and judgment of driver while driving resulting in crashes.^24^

The findings of this study revealed that unlicensed motorists had lower control errors, traffic violations and crash rates than licensed riders. Similar results were found by Bui et al.^22^ Contrary to our results, some research did not approve our results.^25,26^ According to an umbrella review, there was a substantial decrease in the frequency of crashes among 16 and 17-year-old in the United States after implementing driver's license.^27^ An explanation for this difference might be that motorists without a riding license are more careful when riding because they are concerned about being penalized by the traffic police. Future research on the efficiency and effectiveness of the motorcycle training and licensing system is recommended.^28^

Consistent with previous studies of crash risk factors (9, 29), our SEM model estimates attested a negative effect between age and speed violations, stunts, and traffic violation. For example, these studies found that traffic violation (call or text message while riding and cross junction when traffic light is red) emerges more among young riders than in other age groups.^7,9,11-18,21-27,29-34^ In addition, Stephens et al in 2017 concluded that older riders reported less stunt, speed violation, and traffic violation behavior than riders aged 26–39 years and have less crash involvements. Also, our findings revealed that older adults are more likely to exert safety violations than young people. It may be because older motorcyclists are more concerned about their safety than young people. According to the findings of this study, there is a negative association between age and traffic errors and control errors. It might be because younger riders are inexperienced in riding than older people, so they make more errors. On the other hand, the majority of our participants were young adults aged 18-50 and only a small percentage of riders were 50 years and older (2.7%). It seems that in our study, the impact of experience is more evident, so with rising experience, control error and traffic error had decreased. 

The frequency of riding has a significant relationship with the components of traffic error, speed violations, traffic violations, safety violations, and crash experience. The potential reason relates directly to exposure. For instance, more frequent and more prolonged usage of motorcycles is associated with higher errors, violations, and crash rates. Those who ride only a few times a year reported very low crash experiences, whereas those who ride daily had the highest involvement. 

This study found that speed violation, stunt, traffic violation, and safety violations significantly affect the history of crashes as an independent risk factor for collisions. These results reinforce the findings of studies show that speed violations became an influential factor in 20% of fatal crashes and 9% of crash injuries in Spain in 2015.^4^ Some evidence also suggests that stunts can aid in predicting transition to the crash involvements.^7,9^ Furthermore, riders who reported frequent stunt behaviors had three-fold odds of collision than motorcyclists who reported the least frequent stunt behaviors.^5^

Whilst traffic error scale was obtained as the main predictor of accidents in English and Indian motorcyclists,^4,32^ speed violation was the most predictor of self-reported crashes in the present study, and there was no significant relationship between traffic error and crash history. 

According to this study, raising safety violations reduces traffic injuries. No immediately obvious reasons for this correlation were identified. Wearing helmets may encourage motorists to take more risks or take less care when riding, and more likely to have traffic crash. The explanation for the lack or rare similar publications with this conclusion is because in countries where research on motorcycle rider behavior has been performed, wearing a helmet is evident. Thus, even in the MRBQ questionnaire done by Elliott et al in 2007,^4^ there are no questions about the use of helmets by motorcyclists. While in the validated study of MRBQ by Motevalian et al. in Iran, this question was included to the component of safety violations.^10^

Despite the highest motorcyclists in Iran and other countries such as Thailand, Indonesia, and Malaysia, the use of personal protective clothing is extremely uncommon in these countries.^7,19,33^ Thus, similar to findings in these studies, it seems that although the number of motorcycle-related crashes is abnormally high, Vietnamese motorists stated that they are not yet in the habit of using safety equipment during riding.^34,35^ This can be explained by the fact that, aside from the mandatory use of helmets, there are no restrictions on the usage of motorcycle protective clothing in mentioned countries. 

In general, the present study also showed that violations are more effective than errors in crash involvements. These results were not consistent with a study by Elliott et al,^4^ but was consistent with a study by Reason et al^8^ which examined the driving behavior of car users.

**Strengths and limitations**

The strengths of the study include a large sample of riders (n=1,611), giving a great statistical power to our analysis. Also, the data were collected from three cities, so it might be representative of the target population. Inevitably, this study has some limitations such as the cross-sectional design in which the possibility of establishing causal relationships is sophisticated and can only explain the correlation between factors. Additionally, the data related to risky riding behavior were extracted from self-reported measures, so individuals’ responses could be influenced due to memory and social desirability biases. However, Lajunen et al. in 2003 suggested that self-reported riding behaviors are relatively reliable and free from social desirability bias.^36^

## Conclusion

According to the results, age and riding frequency from sociodemographic characteristics are the main variables that affect crash involvement through speed violation, stunt, traffic violation and safety violation. This is an indication that younger riders should avoid speedy rid-ing and risky behaviors, which greatly reduces crashes. Stricter regulations such as training courses should also be considered in traffic safety laws. 
